# DNA polymerase ε leading strand signature mutations result from defects in its proofreading activity

**DOI:** 10.1016/j.jbc.2023.104913

**Published:** 2023-06-10

**Authors:** Robert E. Johnson, Louise Prakash, Satya Prakash

**Affiliations:** Department of Biochemistry and Molecular Biology, University of Texas Medical Branch at Galveston, Galveston, Texas, USA

**Keywords:** DNA polymerase ε, DNA polymerase δ, DNA polymerase ε defects in proofreading, DNA polymerase ε signature mutations, DNA polymerase ε role

## Abstract

The evidence that purified pol2-M644G DNA polymerase (Pol)ε exhibits a highly elevated bias for forming T:dTTP mispairs over A:dATP mispairs and that yeast cells harboring this Polε mutation accumulate A > T signature mutations in the leading strand have been used to assign a role for Polε in replicating the leading strand. Here, we determine whether A > T signature mutations result from defects in Polε proofreading activity by analyzing their rate in Polε proofreading defective *pol2-4* and *pol2-M644G* cells. Since purified pol2-4 Polε exhibits no bias for T:dTTP mispair formation, A > T mutations are expected to occur at a much lower rate in *pol2-4* than in *pol2-M644G* cells if Polε replicated the leading strand. Instead, we find that the rate of A > T signature mutations are as highly elevated *in pol2-4* cells as in *pol2-M644G* cells; furthermore, the highly elevated rate of A > T signature mutations is severely curtailed in the absence of PCNA ubiquitination or Polζ in both the *pol2-M644G* and *pol2-4* strains. Altogether, our evidence supports the conclusion that the leading strand A > T signature mutations derive from defects in Polε proofreading activity and not from the role of Polε as a leading strand replicase, and it conforms with the genetic evidence for a major role of Polδ in replication of both the DNA strands.

The “division of labor” model and designation of DNA polymerase (Pol) ε as the leading strand replicase and of Polδ as the lagging strand replicase has been derived from studies involving mutator alleles of yeast Polε and Polδ and their effects on the distribution of leading or lagging strand mutations. For instance, yeast cells harboring the Polε *pol2-M644G* allele, whose encoded polymerase generates dTTP:T mispairs with an ∼40-fold bias over dATP:A mispairs, exhibit an increased incidence of spontaneous A to T signature mutations in *URA3* integrated near ARS306 (1) that can be ascribed to T:dTTP mispair formation in the leading strand. A similar study with the Polδ *pol3-L612M* allele indicated the prevalence of lagging strand signature mutations consistent with the mispair formation bias exhibited by this Pol3 allele (2). However, in extensive genetic studies in different yeast strains, we subsequently provided evidence contradictory to the “division of labor” replication model, wherein L612M-Polδ generated errors occur on both the leading and lagging DNA strands in *pol3-L612M msh2Δ* strains (3). We postulated that a more proficient removal of errors by mismatch repair (MMR) from the leading strand accounts for the lack of L612M-Polδ specific errors on this strand and concluded from these studies that Polδ replicates both the leading and lagging DNA strands (3).

The four subunit yeast Polε holoenzyme is comprised of the Pol2 catalytic subunit and the Dpb2, Dpb3, and Dpb4 accessory subunits. While Dpb3 and Dpb4 are not essential (4, 5), deletion of either the Pol2 or Dpb2 subunits leads to cell inviability (6, 7). Within the Pol2 protein, the N-terminal half encompasses the active polymerase and the extreme C-terminus harbors a zinc-finger motif that is involved in binding the Dpb2 subunit. Importantly, the essential role of Pol2 lies in its ability to bind Dpb2, whereas the N-terminal catalytic polymerase domain of Pol2 is dispensable, although cells grow slowly (8). The Dpb2 subunit also binds directly to GINS (9, 10), a component of the CMG helicase that encircles and travels on the leading strand in the 3′→5′ direction, unwinding the replication fork. Thus, *via* assembly of the CMG complex, the Pol2 C-terminus plays an essential role in replication by promoting origin firing and DNA unwinding (9, 11, 12).

Extrapolating from our genetic evidence that Polδ replicates both the leading and lagging DNA strands (3), we hypothesized that leading strand A > T signature mutations in *pol2-M644G* reflect Polδ misinsertions which escape proofreading by Polε 3’→5′ exonuclease. To verify this hypothesis, in this study, we determine the rate of A > T signature mutations in Polε proofreading defective *pol2-M644G* and *pol2-4* mutants wherein the *pol2-4* mutation abolishes Polε proofreading, and the *pol2-M644G* mutation impairs mispair recognition (13) rendering proofreading ineffective. However, compared to the highly elevated bias of purified pol2-M644G Polε for forming T:dTTP mispair over the reciprocal A:dATP mispair, purified pol2-4 Polε exhibits no bias for T:dTTP mispair formation (14, 15). Hence, if A > T signature mutations in the leading strand resulted from the role of Polε as a leading strand replicase, A > T signature mutations would occur at a much lower rate in *pol2-4* cells than in *pol2-M644G* cells. However, if A > T signature mutations were derived from a role of Polε proofreading activity, then these mutations would occur at nearly the same rate in the *pol2-4* strain as in *pol2-M644G.* Furthermore if A > T signature mutations were due to Polε role in leading strand replication, then there would be no need for the PCNA ubiquitination-dependent recruitment of Polζ for their formation—given the very high proficiency of pol2-M644G Polε for promoting synthesis from T:dTMP mispairs. Our evidence that A > T signature mutations in *URA3* occur at the same rate in *pol2-M644G* and *pol2-4* strains and that PCNA ubiquitination and Polζ are required for their formation supports the conclusion that the prevalence of leading strand-specific mutations does not arise from a role of Polε in replication of this strand; rather, it derives from the role of Polε proofreading activity in the removal of Polδ misinsertions on the leading strand.

## Results

### Leading strand signature mutations in pol2-M644G are dependent upon PCNA ubiquitination and Polζ

**Figure 1:**
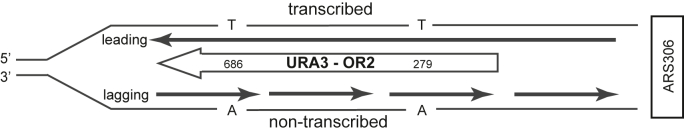
**Schematic representation of the *URA3* forward mutation system**. The *URA3* reporter gene is integrated into chromosome three to the left of ARS306 such that in orientation 2 (OR2), the transcribed strand is replicated as the leading strand, whereas the non-transcribed, coding sequence acts as the lagging strand template. The location of the *URA3* hot spots at positions 686 and 279 nucleotides are shown. The thymine (T) nucleotides present in the leading strand give rise to dTTP:T mispairs in the *pol2-M644G* strain which are identified as A to T mutations in the *ura3* ORF by genomic sequence analysis of FOA resistant colonies.

In both lacZ and steady-state kinetic DNA polymerase fidelity assays, mutant pol2-M644G Polε has been shown to exhibit an ∼ 40-fold bias for the misincorporation of dTTP opposite template T than for the complementary dATP opposite template A (1). Since yeast cells that harbor the *pol2-M644G* mutation exhibit an elevated rate of spontaneously arising A > T hotspot mutations, namely A686T and A279T, in a *URA3* reporter gene when integrated into the antisense orientation (OR2) to the left of ARS306 (1, 2, 3) (Fig. 1); these A > T mutations have been proposed to arise from T: T mispairs formed during replication of the leading strand by Polε. As shown in Table 1, the *pol2-M644G* strain exhibits a *URA3* mutation rate ∼24-fold higher than WT cells. To examine the specific effect on rates of the A686T and A279T signature mutations, we determined the rates of these mutations through sequence analysis of *ura3* mutations arising in a large number of independent cultures. As shown in Table 2, the rate of A > T mutations is extremely elevated in the *pol2-M644G* strain compared to WT (∼1100 fold increase).

**Table 1:** Forward mutation rates of *URA3 (OR2)* to *ura3* in *pol2-M644G* strains

Genotype	5-FOA^r^ rate [×10^−8^] (95% CI)	Rate relative to WT
WT	1.2 (0.8–1.6)	1.0
*rev3Δ*	0.22 (0.18–0.26)	0.2
*pol30–119*	0.9 (0.85–0.95)	0.75
*pol2-M644G*	28.3 (20.6–36.0)	23.6
*pol2-M644G rev3Δ*	7.5 (6.6–8.4)	6.3
*pol2-M644G pol30–119*	6.8 (4.4–9.2)	5.7
*pol2-M644G pol30–119 rev3Δ*	4.9 (4.0–5.8)	4.1

**Table 2:** Rates of A to T hotspot mutations in *URA3* (OR2) conferred by the *pol2-M644G* mutation carried in different genetic backgrounds

Genotype	Total FOA^r^ sequenced	A686T	Rate A686T (×10^−8^)	A279T	Rate A279T (×10^−8^)	Total A→T	Rate A→T (×10^−8^)	Rate relative to WT
WT	55	1	0.02	0	−	1	0.02	1.0
*rev3Δ*	29	0	0	0	0	1	0.008	0.4
*pol30–119*	41	2	0.04	0	0	2	0.04	2.0
*pol2-M644G*	84	47	15.8	12	4.0	66	22.2	1110
*pol2-M644G rev3Δ*	83	51	4.6	3	0.27	60	5.4	270
*pol2-M644G pol30–119*	62	19	2.1	3	0.33	27	3.0	150
*pol2-M644G rev3Δ pol30–119*	100	33	1.6	3	0.15	44	2.2	110

**Figure 2:**
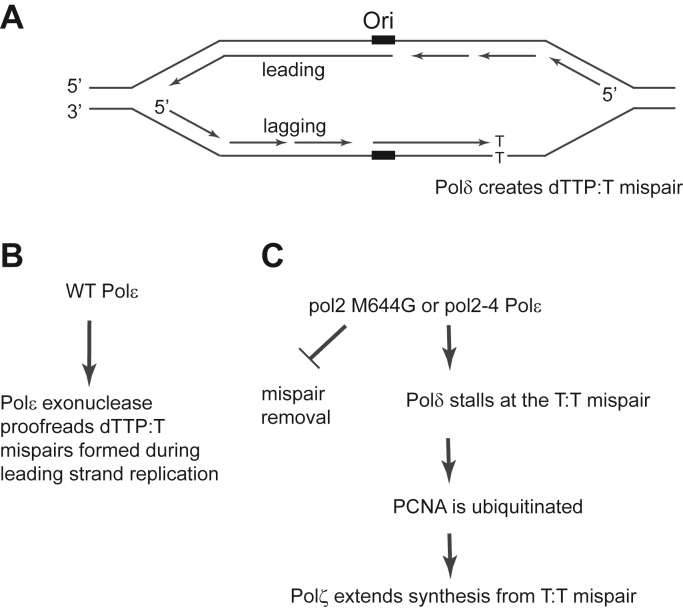
**Role of Pol ε 3′-5′ exonuclease activity in the removal of leading strand Polδ errors.***A*, schematic of a replication fork emanating from a yeast origin (Ori). The leading strand is initiated and replicated by DNA Polδ, whereupon a dTTP:T mispair is generated. If the misinserted nt escapes removal, the replication fork stalls. *B*, in wildtype *Polε* cells, Polε exonuclease proofreads the dTTP:T mispair. *C*, in *pol2-M644G* or *pol2-4* cells, dTTP:T mispairs are not proof read. Polδ stalling at the mispair leads to PCNA ubiquitination and recruitment of Polζ to carry out extension of synthesis from the dTMP:T mispair.

Since Polζ is involved in DNA damage-induced and spontaneous mutation generation (16), and since it is a very proficient extender of synthesis from mispaired termini (17), we next examined whether Polζ was required for spontaneous signature mutations generated in the *pol2-M644G* strain. We find that deletion of the catalytic subunit of Polζ (*rev3Δ*) in *pol2-M644G* cells results in an ∼4-fold reduction in the *URA3* spontaneous mutation rate compared to that in the *pol2-M644G* strain (Table 1). When examined for specific A > T signature mutations, *rev3Δ* reduces the rate of A686T mutations in *pol2-M644G* by ∼ 4-fold, as was the reduction in the overall A > T mutation rate (Table 2). Since PCNA ubiquitination is required for Polζ function in cells (16), we next examined the effect of the *pol30-119* mutation, which harbors an Arg mutation at Lys164 and thus inhibits PCNA ubiquitination (18, 19). Although the overall drop in the spontaneous mutation rate of *URA3* in *pol2-M644G pol30-119* was similar to that found in the *pol2-M644G rev3Δ* strain (Table 1), there was a more pronounced effect on the signature A > T mutations. For instance, signature A686T mutation rates in *pol2-M644G pol30-119* dropped by nearly 8-fold, and the overall rate of A > T mutations was also reduced by ∼ 8-fold in *pol2-M644G pol30-119* (Table 2). When we examined signature mutation rates in *pol2-M644G* cells harboring both the *rev3Δ* and *pol30-119* mutations, the rates were similar to those in the *pol2-M644G pol30-119* strain, indicating that *rev3Δ* and *pol30-119* act epistatically in *pol2-M644G* dependent A > T hotspot mutation formation (Table 2). Altogether, we deduce from our data (Table 2) that the formation of leading strand signature mutations in *URA3* in *pol2-M644G* entails a major PCNA ubiquitination and Polζ dependent pathway (Fig. 2), and suggest that an alternative Polζ and PCNA ubiquitination independent pathway would account for the residual A > T signature mutations that remain in the absence of PCNA ubiquitination or Polζ.

### The exonuclease defective *pol2-4* mutation confers a similar rate of signature mutations as *pol2-M644G*

We and others have previously observed A686T and A279T hotspot mutations occurring in the *URA3*-OR2 reporter gene in strains harboring the *pol2-4* mutation, defective in Polε 3’→5′ proofreading exonuclease (3, 20). This was unexpected since purified Pol2-4 Polε does not exhibit a bias for the generation of dTTP:T mispairs over dATP:A mispairs (14, 15). To examine this further, we determined the rates of A > T signature mutations in the *pol2-4* strain. The spontaneous forward mutation rate in *URA3* in the *pol2-4* strain was ∼44-fold higher than the wild type strain (Table 3). Remarkably, the rate of specific A > T signature mutations was similar to that in the *pol2-M644G* strain. For instance, the rate of A686T formation was 15.8 × 10^−8^ in the *pol2-M644G* strain (Table 2) and 14.3 × 10^-8^ in the *pol2-4* strain (Table 4). The A279T mutation rate in the *pol2-M644G* and *pol2-4* strains was 4.0 × 10^−8^ and 6.0 × 10^−8^, respectively (Tables 2 and 4). Overall, compared to the WT strain, A > T mutations were elevated ∼1100-fold in the *pol2-M644G* strain, and ∼1300-fold in the *pol2-4* strain (Tables 2 and 4).

**Table 3:** Forward mutation rates of *URA3* (OR2) to *ura3* in *pol2-4* strains

Genotype	5-FOA^r^ rate [×10^−8^] (95% CI)	Rate relative to WT
WT	1.2 (0.8–1.6)	1.0
*rev3Δ*	0.22 (0.18–0.26)	0.2
*pol30–119*	0.9 (0.85–0.95)	0.75
*pol2-4*	52.6 (46.4–58.8)	43.8
*pol2-4 rev3Δ*	7.5 (6.4–8.6)	6.3
*pol2-4 pol30–119*	7.9 (3.9–11.9)	6.6
*pol2-4 rev3Δ pol30–119*	6.4 (4.0–8.8)	5.3

**Table 4:** Rates of A to T hotspot mutations in *URA3* (OR2) conferred by the *pol2-4* mutation carried in different genetic backgrounds

Genotype	Total FOA^r^ sequenced	A686T	Rate A686T (×10^−8^)	A279T	Rate A279T (×10^−8^)	Total A→T	Rate A→T (×10^−8^)	Rate relative to WT
WT	55	1	0.02	0	−	1	0.02	−
*rev3Δ*	29	0	0	0	0	1	0.008	0.4
*pol30–119*	41	2	0.04	0	0	2	0.04	2.0
*pol2-4*	88	24	14.3	10	6.0	45	26.9	1345
*pol2-4 rev3Δ*	86	15	1.3	3	0.26	24	2.1	105
*pol2-4 pol30–119*	86	13	1.2	4	0.37	19	1.7	85
*pol2-4 rev3Δ pol30–119*	80	13	1.0	5	0.40	25	2.0	100

### A>T signature mutations in pol2-4 are dependent upon PCNA ubiquitination and Polζ

**Figure 3:**
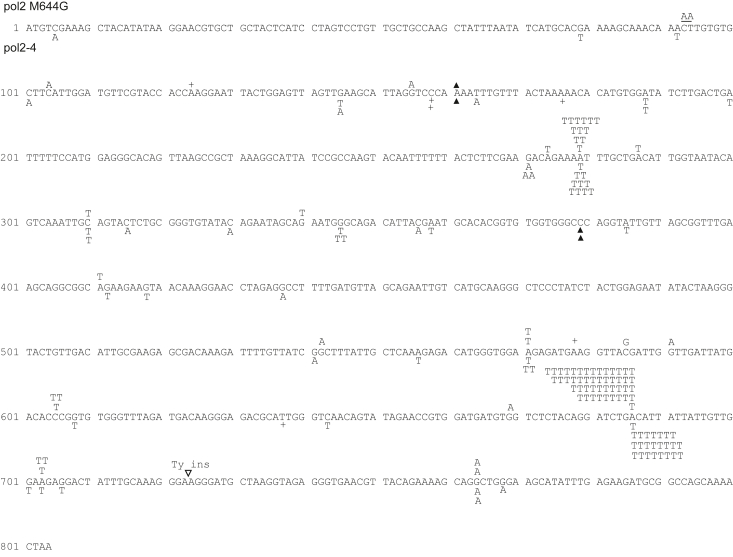
***Ura3* forward mutations arising in *pol2-M644G* and *pol2-4* yeast strains.** The *ura3* gene from multiple FOA-resistant colonies, each arising from an independent culture, was sequenced as described in the Experimental procedures. Mutations arising in yeast strain YPO684 (*pol2-M644G*) are shown above the sequence, and those arising in strain YPO-735 (*pol2-4*) are shown below the sequence. Base substitutions are indicated by G, A, T, or C above or below the WT *POL2* sequence. Single base pair additions and deletions are indicated by a + or a ▲, respectively.

**Figure 4:**
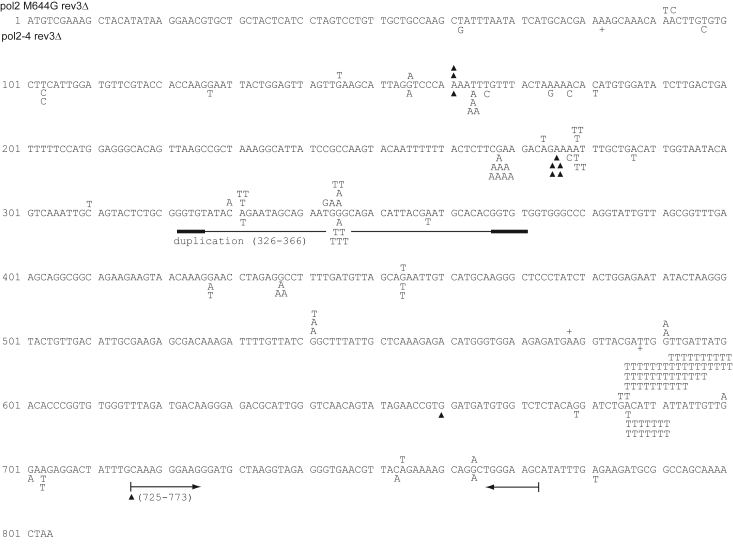
***Ura3* forward mutations arising in *pol2-M644G rev3Δ* and *pol2-4 rev3Δ* yeast strains.** Mutations were identified and depicted as indicated in Figure 3. Multiple base deletions are indicated by a ▲ with a bar spanning the deleted residues. Large duplications and deletions are indicated by solid lines with the corresponding duplicated or deleted residue numbers in parentheses. *Bold lines* indicate regions of homology flanking the duplications and deletions. Mutations arising in yeast strain YPO784 (*pol2-M644G rev3Δ*) are shown above the sequence, and those arising in strain YPO-782 (*pol2-4 rev3Δ*) are shown below the sequence.

**Figure 5:**
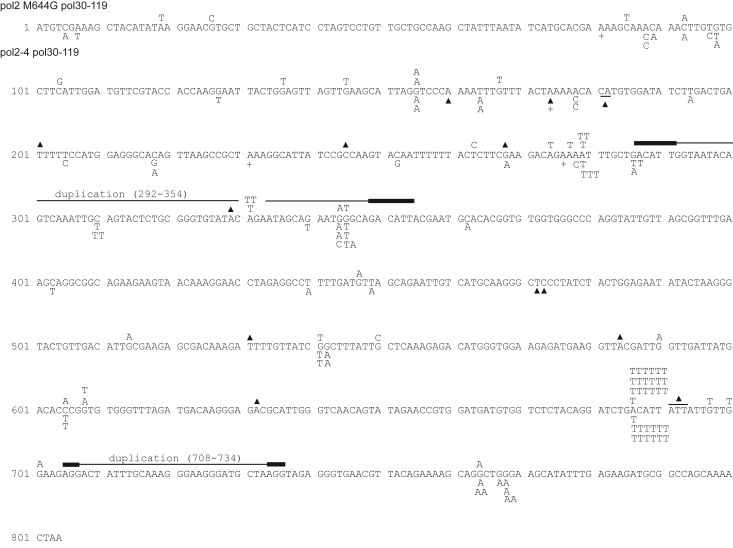
***Ura3* forward mutations arising in *pol2-M644G pol30-119* and *pol2-4 pol30-119* yeast strains.** Mutations were identified and depicted as indicated in Figure 4. Mutations arising in yeast strain BJY364 (*pol2-M644G pol30–119*) are shown above the sequence, and those arising in strain BJY415 (*pol2-4 pol30–119*) are shown below the sequence.

Since the formation of *pol2-M644G* dependent A > T signature mutations requires PCNA ubiquitination and Polζ, we next examined whether PCNA ubiquitination and Polζ were also required for *pol2-4* dependent signature mutations. As shown in Table 3, the spontaneous *URA3* forward mutation rate in *pol2-4* was lowered ∼ 7 to 8-fold by either the *rev3Δ, pol30-119*, or the *rev3Δ pol30-119* double mutation. The overall rate of A > T mutations dropped by ∼13-fold in the *pol2-4 rev3Δ pol30-119* strain, similar to that in the *pol2-4 rev3Δ* or in the *pol2-4 pol30-119* strains (Table 4). Our results that the overall rate of A > T mutations in the *pol2-4 rev3Δ pol30-119* strain is reduced to the same extent as in the *pol2-4 rev3Δ* or *pol2-4 pol30-119* strains concur with an epistatic interaction of *rev3Δ* with *pol30-119* in *pol2-4* Polε dependent mutation generation (Table 4). Altogether, we infer from these data that A > T signature mutation formation observed in the *pol2-4* strain occurs *via* a pathway involving PCNA ubiquitination and Polζ (Fig. 2); and another pathway that operates independently of PCNA ubiquitination and Polζ would account for the mutations that remain. The sequence data for the various strains are shown in Figure 2, Figure 3, Figure 4, Figure 5.

**Figure 6:**
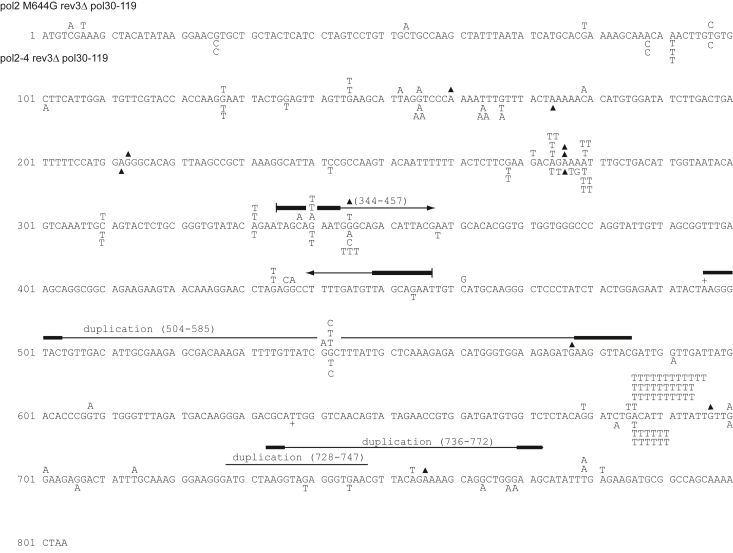
***Ura3* forward mutations arising in *pol2-M644G rev3Δ pol30-119* and *pol2-4 rev3Δ pol30-119* yeast strains.** Mutations were identified and depicted as indicated in Figure 4. Mutations arising in yeast strain BJY580 (*pol2-M644G rev3Δ pol30–119)* are shown above the sequence, and those arising in strain BJY601 (*pol2-4 rev3Δ pol30–119)* are shown below the sequence.

## Discussion

### Signature mutations in pol2-M644G do not signify Pol**ε** role in leading strand replication

Polε has been implicated as the leading strand replicase, in part from the evidence that the elevated rate of A > T signature mutations observed in *pol2-M644G* yeast strains correlates with an extreme bias of M644G Polε for the formation of dTTP:T mispairs that would occur in the leading strand. During replication, M644G Polε would therefore have a high propensity for dTTP:T mispair formation and for proficiently extending synthesis from those mispairs, rather than proofread them. However, we find that these signature mutations are Polζ-dependent and they require ubiquitination of PCNA. If A > T mutations were generated by *pol2-M644G* Polε as the leading strand replicase *via* the formation and extension of synthesis from dTMP:T mispairs, then there would have been no need for Polζ. Thus, by that measure, *i.e.* the formation of leading strand signature mutations, the requirement of Polζ would suggest that it too is a major replicase for the leading strand, which it is not. Furthermore, the reduction in *URA3* signature mutations by *pol30-119* implies that their formation depends upon the ubiquitination of PCNA, a process not required for replication of the leading strand. Thus, the high incidence of spontaneously arising A > T signature mutations in the *pol2-M644G* yeast strain is not an indicator of the role of Polε as the major leading strand replicase.

### Leading strand signature mutations result from lack of removal of Polδ misinsertions in the absence of proofreading by Pol**ε**

Remarkably, the yeast *pol2-4* mutation confers a nearly identical increase in the rate of A > T signature mutations in the *URA3* reporter gene as the *pol2-M644G* mutation. Thus, the A > T mutations in *pol2-M644G* cells which were thought to have resulted from the 40-fold bias of M644G Polε for dTTP:T mispair formation (1) arise at the same high rate in *pol2-4* cells, despite the fact that this exonuclease deficient polymerase exhibits no bias for generating dTTP:T mispairs (14, 15). Hence, these *pol2-4* dependent leading strand-specific A > T signature mutations in *URA3* must derive from a process that is not dependent upon Polε mispair insertion, but are rather dependent upon the lack of removal of dTTP:T mispairs already present in the leading strand. The only way to explain these results is that A > T mutations in *pol2-M644G* and *pol2-4* cells derive from a major role of Polδ in the replication of the leading strand (3), and that they reflect Polδ mis-insertions which escape proofreading by its own 3’→5′ exonuclease and which are recalcitrant to removal by MMR (21). Thus, A > T signature mutations would accumulate on the leading strand in these Polε mutants because of the reduced ability of *pol2-M644G* Polε to recognize (13) and the inability of *pol2-4* Polε to proofread such Polδ generated mispairs, and not because mutant Polε generates dTTP:T mispairs at a high rate during replication.

### Somatic Pol**ε** proofreading domain mutations in cancers

The conclusions of this study imply that the high prevalence of mutations that occur in a large variety of cancers harboring somatic Polε proofreading domain mutations (22, 23, 24, 25, 26, 27, 28, 29) derive from PCNA ubiquitination and Polζ dependent extension of synthesis from Polδ generated mispairs on the leading strand that do not get removed in the absence of Polε proofreading function. Furthermore, the indispensability of Polδ for replication of both the DNA strands (3) explains the dearth of somatic Polδ proofreading domain mutations; and the requirement of Polε proofreading activity for the removal of specific Polδ generated mispairs on the leading strand explains the high prevalence of somatic Polε proofreading domain mutations that occur in cancer genomes (29).

### Dispensability of Polε polymerase activity for viability

In striking contrast to the indispensability of Polδ polymerase activity for viability (30, 31, 32, 33), the lack of N-terminal Polε polymerase domain supports viability, although cell growth is affected (8). Nevertheless, the observation that the lethality of *pol2Δ* cells is efficiently rescued by the *pol2* mutation that is defective in its polymerase activity and in its PCNA binding PIP domain (34) reinforces the dispensability of Polε polymerase activity for cell survival. These results and the evidence that Polδ signature mutations occur on both DNA strands in *pol3-L612M msh2Δ* (3, 35) and that defects in Polε proofreading activity account for Polε leading strand signature mutations in *pol2-M644G* or *pol2-4* cells (this study) can be explained only if Polδ replicated both the DNA strands and Polε contributed primarily to DNA repair roles on the leading strand.

## Experimental procedures

### Yeast strains

All genetic experiments were carried out in isogenic derivatives of the S288C-based yeast strain BY4741 (*MAT***a** *his3Δ1 leu2Δ0 met15Δ0 ura3Δ0)* (36). The *pol2-4* and *pol2-M644G* mutations were integrated into the yeast genome by direct replacement of the wild-type *POL2* gene using either pPOL550 or pPOL520, respectively (3). The *pol2-pip* (FF1199,1200AA) mutation was generated by PCR using mutagenic oligonucleotides, and the resulting PCR fragment was subcloned into the Pol2 direct replacement vector, generating pPOL551. The *pol2-M644G, pip* double mutant replacement plasmid, pPOL779, was constructed similarly. Yeast strains harboring the *pol2 M644G, pol2 pip,* and *pol2-M644G pip* mutations were generated by transformation with the respective plasmids digested with FspI/SwaI restriction endonucleases, and selected for growth on synthetic complete (SC)-uracil media. Excision of the *URA3* selectable marker integrated into the 5′ UTR of *pol2* was selected by plating on media containing 5-fluoro-orotic acid (FOA) and confirmed by PCR analysis of yeast genomic DNA. To generate yeast harboring the *pol2-4 pip* double mutation, the *pol2 pip* yeast strain YPO-861 was transformed with pPOL550 digested with EcoRI, which integrates the *pol2-4* mutation while leaving the *pol2 pip* mutation intact. The *rev3Δ* mutation was generated by transformation with plasmid pRev3.75 digested with EcoRI/BamHI and the *pol30-119* mutation was integrated into the genome by gene replacement with plasmid pPCNA1.44 digested with Asp718/XbaI. Loss of the *URA3* geneblaster was selected by plating cells on 5-FOA media. All genomic mutations were confirmed by either restriction enzyme digestion and/or by sequence analysis of PCR products amplified from yeast genomic DNA.

### URA3 forward mutation analysis

To monitor spontaneous forward mutations of *URA3* integrated near ARS306, the various yeast strains were transformed to *URA3*^*+*^ with pBJ2176 digested with XhoI/SalI, which targets the integration of the *URA3* gene in the antisense orientation (OR2) ∼1100 bp to the left of ARS306, between the *FUS1* and *HBN1* genes, in chromosome 3. We previously showed that integration of *URA3* at this genomic position in the yeast genome does not alter the firing of ARS306 (3).

### *URA3* to *ura3* mutation rates and spectra

Spontaneous forward mutation rates of *URA3* OR2 were determined for each yeast strain using the method of the median (37). For each strain, 9 to 15 independent cultures, each starting from ∼100 *URA3+* cells were grown in 3 ml of YPD medium for 3 days. Cells were sonicated, harvested by centrifugation, and then washed and resuspended in sterile water. To determine the median number of mutations arising in the cultures, appropriate cell numbers were plated on SC complete media containing 5-FOA. To determine cell culture viability, appropriate dilutions were plated on SC complete media (Sunrise Science Products). Experiments were repeated 3 to 4 times. For sequence analyses, additional independent cultures were grown as described above, washed, and plated on media containing 5-FOA. A single FOA^r^ colony arising from each culture was patched onto YPD and genomic DNA was extracted. The *ura3* gene was amplified *via* PCR and the products were sequenced using oligos LP2221 and LP2222 (3).

### Data availability

All of the study data are included in the article.

## Conflict of interest

The authors declare that they have no conflicts of interest with the contents of this article.
